# Transcranial direct current stimulation as a memory enhancer in patients with Alzheimer’s disease: a randomized, placebo-controlled trial

**DOI:** 10.1186/s13195-016-0180-3

**Published:** 2016-03-23

**Authors:** Martin Bystad, Ole Grønli, Ingrid Daae Rasmussen, Nina Gundersen, Lene Nordvang, Henrik Wang-Iversen, Per M. Aslaksen

**Affiliations:** Department of Psychology, Research Group for Cognitive Neuroscience, Faculty of Health Sciences, University of Tromsø, Tromsø, Norway; Department of Child and Adolescent Psychiatry, University Hospital of North Norway, Tromsø, Norway; Department of Geropsychiatry, University Hospital of North Norway, Tromsø, Norway

**Keywords:** Alzheimer’s disease, Randomized controlled trial, Transcranial direct current stimulation, Memory, Neuropsychology, Neuromodulation

## Abstract

**Background:**

The purpose of this study was to assess the efficacy of transcranial direct current stimulation (tDCS) on verbal memory function in patients with Alzheimer’s disease.

**Methods:**

We conducted a randomized, placebo-controlled clinical trial in which tDCS was applied in six 30-minute sessions for 10 days. tDCS was delivered to the left temporal cortex with 2-mA intensity. A total of 25 patients with Alzheimer’s disease were enrolled in the study. All of the patients were diagnosed according to National Institute of Neurological and Communicative Disorders and Stroke and Alzheimer’s Disease and Related Disorders Association criteria. Twelve patients received active stimulation, and thirteen patients received placebo stimulation. The primary outcome measure was the change in two parallel versions of the California Verbal Learning Test–Second Edition, a standardized neuropsychological memory test normalized by age and gender. The secondary outcome measures were the Mini Mental State Examination, clock-drawing test, and Trail Making Test A and B.

**Results:**

Changes in the California Verbal Learning Test–Second Edition scores were not significantly different between the active and placebo stimulation groups for immediate recall (*p* = 0.270), delayed recall (*p* = 0.052), or recognition (*p* = 0.089). There were nonsignificant differences in score changes on the Mini Mental State Examination (*p* = 0.799), clock-drawing test (*p* = 0.378), and Trail Making Test A (*p* = 0.288) and B (*p* = 0.093). Adverse effects were not observed.

**Conclusions:**

Compared with placebo stimulation, active tDCS stimulation in this clinical trial did not significantly improve verbal memory function in Alzheimer’s disease. This study differs from previous studies in terms of the stimulation protocol, trial design, and application of standardized neuropsychological memory assessment.

**Trial registration:**

ClinicalTrials.gov identifier NCT02518412. Registered on 10 August 2015.

## Background

Neuroimaging studies have suggested that Alzheimer’s disease is associated with pathological and structural changes in the brain, especially in the temporal cortex [1]. Several studies have demonstrated that stimulation of the temporal cortex with transcranial direct current stimulation (tDCS) can enhance name recall in healthy elderly persons [2] and improve recognition memory in patients with Alzheimer’s disease [3–5]. tDCS is noninvasive and works by inducing a low direct current in the cortical area of interest [6]. Small electrodes are placed on the scalp above the brain area that is targeted by tDCS. This stimulation facilitates cortical excitability and thereby neuroplasticity [6].

The results of previous studies are promising [3–5]. However, there is still insufficient evidence that supports tDCS as an intervention for Alzheimer’s disease. Randomized, placebo-controlled trials are warranted to assess the efficacy of temporal cortex tDCS in patients with Alzheimer’s disease. Trials should include more comprehensive outcome measures to explore the effect of tDCS on memory function. The aim of the present study was to investigate the effect of tDCS on verbal memory functions in patients diagnosed with Alzheimer’s disease.

## Methods

### Study design and participants

A randomized, placebo-controlled trial with a parallel group design was performed. Two groups were included in the intervention: an active tDCS group and a placebo tDCS group. The allocation ratio was 1:1.

Patients diagnosed with Alzheimer’s disease were invited to participate in the study via a letter from the Department of Geriatric Medicine at the University Hospital of North Norway, and healthy participants were recruited through a newspaper advertisement. The eligibility criteria were living at home and fulfillment of the research criteria for the likelihood of having Alzheimer’s disease according to the revised criteria of the National Institute of Neurological and Communicative Disorders and Stroke and Alzheimer’s Disease and Related Disorders Association criteria [7]. We followed section 4.2 in these criteria: “Probable Alzheimer’s disease with increased level of certainty.” This determination of eligibility for the study requires evidence of a progressive cognitive decline based on information from informants (relatives) and a cognitive and/or neuropsychological evaluation [7].

We excluded patients who scored <18 on the Mini Mental State Examination (MMSE) [8]. Other exclusion criteria included serious somatic disorders (cancer, chronic obstructive pulmonary disease, and heart failure) or neuropsychiatric disorders (e.g., severe depression and psychosis) that might reduce cognitive abilities. The patients with comorbid cerebral conditions, such as cerebrovascular injuries and/or stroke, brain tumor, or Parkinson’s disease, were not eligible to participate in the study. Patients using cholinesterase inhibitors had to have been using them for at least 3 months before enrolling in the study. A total of 25 patients with Alzheimer’s disease were included in the study.

A total of 22 healthy elderly volunteers, aged 59–83 years, served as controls for the neuropsychological test performance at baseline. None of them had cognitive impairment or other serious diseases. These healthy volunteers were recruited through an advertisement. The control group did not receive any tDCS stimulation. They completed the Hospital Anxiety and Depression Scale [9], a questionnaire used to screen for depression and anxiety.

**Fig. 1:**
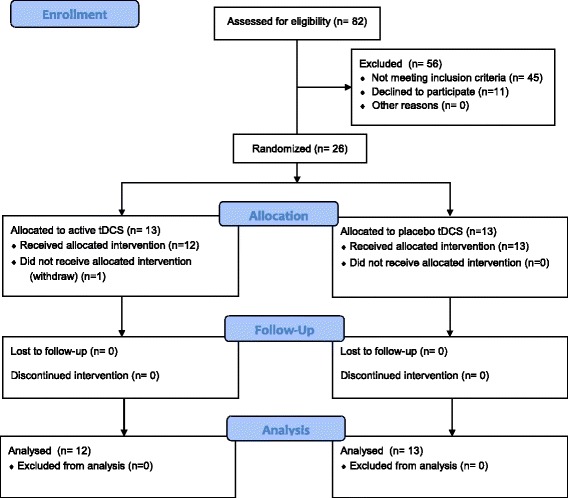
Flow diagram of trial profile

The neuropsychological test battery used for healthy volunteers and patients with Alzheimer’s disease was identical. The study was executed in a research laboratory at the University of Tromsø Institute of Psychology. The study was ethically approved by the regional committee for medical and health research ethics (2012/1890) and was registered in the ClinicalTrials.gov database with the identifier NCT02518412. All of the patients and healthy control subjects signed a written informed consent form in line with the Declaration of Helsinki before participating in the study. Each patient received a gift card worth 600 NOK (67 EUR, 75 USD) for their participation. Figure 1 contains a flow diagram of the trial.

### Outcome measures

The primary outcome measure was verbal memory function. We used a validated and standardized Norwegian version of the California Verbal Learning Test–Second Edition (CVLT-II) to assess three aspects of verbal memory function: immediate recall, delayed recall, and recognition [10]. CVLT-II is normed by age and gender and is widely used to assess patients with Alzheimer’s disease [10]. To reduce test-retest effects, the CVLT-II consists of two parallel versions: the CVLT-II standard and alternate forms, which contain two different and independent word lists. We used the standard form at baseline and the alternative form in the posttest.

The secondary outcome measures included the MMSE, clock-drawing test, and Trail Making Test parts A and B (TMT A and B). The MMSE is a screening tool used for assessing cognitive impairment (e.g., orientation, recall, arithmetic, language, and ability to follow simple instructions) [8]. The clock-drawing test is another screening tool used for detecting cognitive impairment and is also used to assess visuoconstructive ability [11]. The TMT consists of part A and part B. TMT A measures sustained attention, whereas TMT B assesses executive function [12].

To control for general cognitive abilities, we used the Wechsler Abbreviated Scale of Intelligence with the matrix reasoning and vocabulary subtests [13]. To screen for depressive symptoms, we used the Cornell Scale for Depression in Dementia [14], which is a questionnaire completed by an informant (i.e., a relative). A score above 13 indicates depression, which was an exclusion criterion in the present study. We documented progressive decline using the Informant Questionnaire on Cognitive Decline in the Elderly (IQCODE) [15], which was also completed by an informant. To assess for potential confusion during neuropsychological testing, the Confusion Assessment Method [16] was applied by a research assistant. This questionnaire is based on the observation of core symptoms of confusion (e.g., inattention, disorganized thinking, and altered level of consciousness).

### Intervention

The intervention was treatment with tDCS using a direct current stimulator (neuroConn, Ilmenau, Germany), which is battery-driven and delivers a direct current. The current intensity was 2 mA, and the stimulation duration was 30 minutes. A pair of 35-cm^2^ rubber electrodes transferred the direct current. These electrodes were inserted into sponge pads soaked with 10 ml of sterile water. To stimulate the left temporal lobe, the anode (positive electrode) was placed at the T3 position in the 10–20 system for electroencephalographic electrode positioning. The cathode (negative electrode) was placed on the right frontal lobe at the Fp2 position. For the placebo tDCS, the electrode placement and session duration were identical to those for active tDCS. However, in the placebo tDCS, the current was delivered for 30 seconds at the beginning of the stimulation, then the current was turned off automatically.

### Randomization and blinding

The patients were assigned to a list with five-digit codes provided by the manufacturer of the tDCS stimulator. Each patient had his or her own code. The codes instructed the stimulator to deliver either placebo or active stimulation. The order of the codes was randomized using the Random.org website (https://www.random.org/). To ensure double-blinding, the list of code assignments was not disclosed during the entire tDCS intervention. The list was decoded when the study was completed to identify the patients in the active and placebo groups. The tDCS stimulator did not display information that could be used to identify the placebo or active stimulation.

### Procedure

**Fig. 2:**
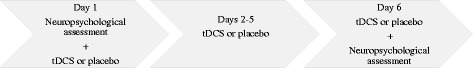
Overview of the procedure

After their inclusion in the study, the patients and their relatives visited the research laboratory and received information regarding the project. During this meeting, the patient completed an informed consent form. Subsequently, the patient underwent neuropsychological testing (baseline). The neuropsychological assessment lasted for approximately 60 minutes, including several short breaks. After the neuropsychological assessment was completed, the first tDCS stimulation commenced. Each patient underwent six sessions of tDCS or placebo tDCS stimulation for 10 days. Each tDCS stimulation session lasted 30 minutes. An experienced research assistant administered the tDCS stimulation. When the last tDCS stimulation was completed, the patient performed the neuropsychological posttesting and received a gift certificate. Figure 2 gives an overview of the procedure.

### Power and statistical analyses

In previous studies in which tDCS was used to stimulate memory functions in patients with Alzheimer’s disease, researchers reported significant results (*p* < 0.05) with a total of ≤15 patients [3–5] in a within-group design. Thus, we aimed to include a larger sample than those described in previous studies [3–5] to ensure accurate analysis of the effects of the intervention.

We used IBM SPSS version 22 software (IBM, Armonk, NY, USA) to perform the statistical analysis. Because of a violation of the assumption of a normal distribution, a nonparametric Mann-Whitney *U* test was conducted to compare the placebo tDCS and active tDCS groups at baseline. A nonparametric Kruskal-Wallis test was used to assess the baseline characteristics for all three groups (placebo tDCS, active tDCS, and healthy control subjects at baseline).

For the primary analyses, the data had a normal distribution. However, because of a small sample size and a large variance, we decided to use a nonparametric Mann-Whitney *U* test for the analysis. With the Mann-Whitney *U* test, we examined the change from baseline to posttest. The raw scores for the neuropsychological tests (CVLT-II and WASI) were scaled according to standardized norm tables [13, 17]. The significance level was set at *p* < 0.05.

## Results

A total of 82 patients diagnosed with Alzheimer’s disease were assessed for eligibility. Of these patients, 45 were excluded because of comorbid and serious somatic diseases, MMSE score <17, and psychiatric diseases. A total of 11 patients declined to participate in the study. One patient decided to withdraw from the study. Twenty-five patients were enrolled in the study and completed the intervention between June 2013 and June 2015. Table 1 shows the patients’ baseline characteristics.

**Table 1:** Baseline characteristics

	Active tDCS (*n* = 12)	Placebo tDCS (*n* = 13)	*p* Value	Controls (*n* = 22)	*p* Value
Age, years	70.0 (8.0) *70.5 (21.0)*	75.0 (8.7) *75.0 (30.0)*	0.12	68.8 (6.8) *69.0 (24.0)*	0.062
Males	7 (58 %)	7 (53 %)	0.85	4 (18 %)	
DM	12 (100 %)	12 (92 %)	0.76		
CVLT-II IR	25 (7.9) *22.0 (25.0)*	23 (6.8) *23.0 (22.0)*	1.00	52.7 (10.0) *54.0 (33.0)*	0.01^a^
CVLT-II DR	−2.7 (0.5) *−2.5 (2.0)*	−2.3 (0.8) *−2.5 (2.5)*	0.4	−0.4 (0.9) *−0.5 (3.5)*	0.01^a^
CVLT-II RG	0.6 (0.9) *0.7 (3.0)*	1.0 (0.5) *1.1 (1.8)*	0.24	1.5 (1.0) *2.4 (3.3)*	0.01^a^
TMT A	91.0 (45.0) *81.0 (138.0)*	143.0 (65.0) *131.0 (191.0)*	0.059	48.5 (18.6) *46.5 (87.0)*	0.01^a^
TMT B	266.0 (123.0) *215.0 (266.0)*	347.0 (225.0) *259.0 (693.0)*	0.67	93.0 (34.8) *90.5 (149.0)*	0 · 01^a^
Clock	3.33 (1.4) *3.5 (5.0)*	1.5 (1.6) *1.0 (4.0)*	0.024^a^	4.86 (0.86) *5.0 (2.0)*	0.01^a^
MMSE	20.0 (2.8) *21.0 (8.0)*	21.2 (3.9) *23.0 (13.0)*	0.71	29.5 (1.09) *30.0 (5.0)*	0.01^a^
WASI Ma	43.0 (9.2) *44.5 (27.0)*	42.5 (6.9) *42.0 (26.0)*	0.81	58.05 (9.0) *61.5 (34.0)*	0.01^a^
WASI Vo	41.7 (9.3) *39.0 (31.0)*	41.6 (14.3) *44.0 (48.0)*	0.76	57.0 (9.9) *57.0 (40.0)*	0.01^a^
Cornell Scale for Depression in Dementia	5.7 (4.3) *6.0 (12.0)*	4.8 (3.4) *5.0 (12.0)*	0.65		
CAM	0.0	0.0	1.0		
IQCODE	3.9 (0.3) *4.1 (1.2)*	4.1 (0.3) *4.2 (1.1)*	1 · 0		

*DM* dementia medications, *CVLT-II IR* California Verbal Learning Test–Second Edition Immediate Recall, *CVLT-II DR* California Verbal Learning Test–Second Edition Delayed Recall, *CVLT-II RG* California Verbal Learning Test–Second Edition Recognition, *WASI* Wechsler Abbreviated Scale of Intelligence, *IQCODE* Informant Questionnaire of Cognitive Decline in the Elderly, *CAM* Confusion Assessment Method, *MMSE* Mini Mental State Examination, *TMT* Trail Making Test, *tDCS* transcranial direct current stimulation

Data are the mean (SD) or n (%). Median and range are displayed in italic type. The first *p* value column shows the differences between the placebo and active groups at baseline. The second *p* value column displays the differences between the active, placebo, and control groups at baseline. For CVLT-II, delayed recall is displayed as age- and gender-adjusted *z*-scores (normalized mean 0, SD 1). For immediate recall the score is displayed as a T-score (normalized mean 50, SD 10), and for recognition the score is an adjusted *d′* score (relationship between total hits and false-positive results). For TMT A and B, results are displayed in seconds. Maximum score on the MMSE is 30. Scores <24 indicate cognitive impairment [8]. Scores on the WASI are displayed as T-scores (normalized mean 50, SD 10). The cutoff score on the IQCODE for Alzheimer’s disease is >3.5 [15]. For the Cornell Scale for Depression in Dementia, a cutoff >12 indicates depression [14]. CAM ranges from 0 to 4, where 0 indicates no symptoms of confusion. The clock-drawing test scores range from 0 to 5, where 5 indicates no errors.

^a^ *p* < 0.05 denotes statistically significant values

In our analysis, we found significant differences between healthy control subjects and patients with Alzheimer’s disease at baseline. Except for the clock-drawing test, there were no significant differences in the baseline characteristics between the placebo and active groups (Table 1).

For the primary outcome measures, scores between the active and the placebo group did not differ significantly on the CVLT-II immediate recall (95 % confidence interval [CI] −9.00 to 2.00; U = 99.00, *z*-score = 1.14, *p* = 0.270, *r* = 0.22), CVLT-II delayed recall (95 % CI −1.0 to 0.0; U = 113.50, *z*-score = 2.132, *p* = 0.052, *r* = 0.42), or CVLT-II recognition (95 % CI −1.25 to 0.18; U = 96.00, *z*-score = 1.38, *p* = 0.089, *r* = 0.27). The scores on the secondary outcome measures (MMSE, clock-drawing test, and TMT A and B) did not differ significantly between the active and placebo tDCS groups (Table 2). Table 3 display the number of patients showing improvement on primary outcome measures.

**Table 2:** Outcome measures

	Active tDCS (*n* = 12)	Placebo tDCS (*n* = 13)	Difference	*p* Value
Primary outcomes				
CVLT-II immediate recall	5.0 (25.0)	0.0 (31.0)	5.0	0.270
CVLT-II delayed recall	0.0 (1.5)	0.0 (2.5)	0.0	0.052
CVLT-II recognition	0.3 (4.0)	−0.08 (1.6)	0.47	0.089
Secondary outcomes				
MMSE	1.0 (9.0)	1.0 (10.0)	0.0	0.799
Clock-drawing test	0.0 (4.0)	0.0 (5.0)	0.0	0.378
TMT A	3.5 (262.0)	−7.0 (219.0)	10.5	0.288
TMT B	22.0 (204.0)	−96.0 (443.0)	118.0	0.093

*CVLT-II* California Verbal Learning Test–Second Edition, *MMSE* Mini Mental State Examination, *TMT* Trail Making Test, *tDCS* transcranial direct current stimulation

Data are the median (range) values. The median values are the estimated change from baseline to posttesting. The positive values indicate positive changes. For the CVLT-II immediate recall, the median value is displayed as a T-score. For the CVLT-II delayed recall, the median value is displayed as a scaled *z*-score. For CVLT recognition, the median value is an adjusted *d′* score. The differences between the placebo and active tDCS were calculated using a nonparametric Mann-Whitney *U* test

**Table 3:** Frequency table

	Active tDCS (*n* = 12)	Placebo tDCS (*n* = 13)
CVLT-II immediate recall	9	6
CVLT-II delayed recall	4	1
CVLT-II recognition	7	4

*CVLT-II* California Verbal Learning Test–Second Edition, *tDCS* transcranial direct current stimulation

The data represent the number of patients showing improvement on primary outcome measures. Improvement was displayed as positive changes from baseline to posttest

### Safety and tolerability

Both patients and their relatives were told to report likely adverse effects (e.g., headache, itching, skin irritation). However, no adverse effects were reported, which indicates that the tDCS intervention was both safe and well-tolerated.

## Discussion

The aim of the present randomized, placebo-controlled study was to assess the effect of tDCS stimulation on verbal memory function in patients with Alzheimer’s disease. We were unable to reveal significant differences between the placebo and active tDCS groups in both primary and secondary efficacy outcomes. We found a tendency for improved delayed recall in the active tDCS group, albeit not significant.

Boggio and colleagues stimulated [4] the temporal cortex in patients with Alzheimer’s disease using a 30-minute tDCS stimulation for 5 consecutive days. This stimulation increased visual recognition memory scores by 8.9 %, and the improvement persisted for 1 month after the last simulation session.

Our results are not in agreement with the results of previous studies [3–5], which can be attributed to several likely explanations. First, we used a fixed stimulation protocol for all patients. Several recent studies suggested that anatomical differences (e.g., skull thickness) can affect current distributions to the cortex [18]. Future tDCS studies will likely take advantage of computational models to ensure individual calibration of the stimulation procedure.

Second, the patients in our study may have been less receptive to tDCS because of the severity of their disease. tDCS stimulation seems to be less effective in the advance stages of Alzheimer’s disease [19, 20]. According to our baseline measures of memory function, a majority of our patients had severe memory impairment (see CVLT-II characteristics in Table 1). Alzheimer’s disease is associated with reduced neuroplasticity (i.e., a considerable reduction in long-term potentiation) [21]. This condition is especially pronounced in the temporal cortex [22] and may inhibit the effect of temporal cortex stimulation when memory impairment is severe.

Third, our study differs from previous studies [3–5] by its limited sample size and in terms of the stimulation procedure, study design, and outcome measures. According to Elder and Taylor [23], different stimulation paradigms should be investigated in Alzheimer’s disease. The optimal stimulation procedure for Alzheimer’s is still uncertain. Thus, the present study is in line with these recommendations and applied a new stimulation paradigm. Clinical application of tDCS is still in its infancy [24]. It is important to find the most effective tDCS paradigm for patients with Alzheimer’s disease.

A major difference between the present study and previous studies [3–5] is our application of standardized memory assessment. This accords with recommendations derived from previous reviews [19, 20]. Neuropsychological testing is considered to be the most reliable method for assessing cognitive function in Alzheimer’s disease [25]. Furthermore, in the present study, we applied a randomized, placebo-controlled design. To the best of our knowledge, this is the first randomized, placebo-controlled study of tDCS stimulation of the temporal cortex in Alzheimer’s disease. Additionally, none of our patients experienced any adverse effects due to the intervention, which indicates that tDCS is safe and well-tolerated.

We recommend future studies with outcome measures that include neuropsychiatric symptoms, neuropsychological assessment, and activities of daily living. The Neuropsychiatric Inventory [26] and the Amsterdam Instrumental Activities of Daily Living Questionnaire [27] are recommended in that regard.

Large-scale randomized controlled studies are warranted. Recruitment is a main barrier. Recruitment presents a challenge for clinical studies of tDCS [18] and trials in Alzheimer’s disease [28]. One way to facilitate the recruitment process is to increase the number of trial sites [28]. In addition, increasing the repetition rate (e.g., stimulation twice per day) could be more feasible and might require fewer separate days of visits to the research laboratory. Such stimulation may even prolong the aftereffects of stimulation [29, 30]. Fewer visits can be beneficial for recruitment [28].

## Conclusions

This randomized, placebo-controlled study failed to reveal any significant results. There was a nonsignificant improvement in delayed recall for the active tDCS condition. This trial showed high tolerability of tDCS. In future research, investigators should use both neuropsychological and neurophysiological outcome measures, study patients in early stages of Alzheimer’s disease, and overcome recruitment barriers to increase power.

## Abbreviations

CAM: Confusion Assessment Method
CI: confidence interval
CVLT-II: California Verbal Learning Test–Second Edition
DM: dementia medications
IQCODE: Informant Questionnaire on Cognitive Decline in the Elderly
MMSE: Mini Mental State Examination
tDCS: transcranial direct current stimulation
TMT: Trail Making Test
WASI: Wechsler Abbreviated Scale of Intelligence

## References

[1] Blennow K, de Leon MJ, Zetterberg H (2006). Alzheimer’s disease. Lancet.

[2] Ross LA, McCoy D, Coslett HB, Olson IR, Wolk DA (2011). Improved proper name recall in aging after electrical stimulation of the anterior temporal lobes. Front Aging Neurosci.

[3] Ferrucci R, Mameli F, Guidi I, Mrakic-Sposta S, Vergari M, Marceglia S (2008). Transcranial direct current stimulation improves recognition memory in Alzheimer disease. Neurology.

[4] Boggio PS, Ferrucci R, Mameli F, Martins D, Martins O, Vergari M (2012). Prolonged visual memory enhancement after direct current stimulation in Alzheimer’s disease. Brain Stimul.

[5] Boggio PS, Khoury LP, Martins DCS, Martins OEMS, de Macedo EC, Fregni F (2009). Temporal cortex direct current stimulation enhances performance on a Visual recognition memory task in Alzheimer disease. J Neurol Neurosurg Psychiatry.

[6] Stagg CJ, Nitsche MA (2011). Physiological basis of transcranial direct current stimulation. Neuroscientist.

[7] McKhann GM, Knopman DS, Chertkow H, Hyman BT, Jack CR, Kawas CH (2011). The diagnosis of dementia due to Alzheimer’s disease: recommendations from the National Institute on Aging-Alzheimer’s Association workgroups on diagnostic guidelines for Alzheimer’s disease. Alzheimers Dement.

[8] Folstein MF, Folstein SE, McHugh PR (1975). “Mini-mental state”: a practical method for grading the cognitive state of patients for the clinician. J Psychiatr Res.

[9] Mykletun A, Stordal E, Dahl AA (2001). Hospital Anxiety and Depression (HAD) scale: factor structure, item analyses and internal consistency in a large population. Br J Psychiatry.

[10] Woods SP, Delis DC, Scott JC, Kramer JH, Holdnack JA (2006). The California Verbal Learning Test – Second Edition: test-retest reliability, practice effects, and reliable change indices for the standard and alternate forms. Arch Clin Neuropsychol.

[11] Shulman KI (2000). Clock‐drawing: is it the ideal cognitive screening test?. Int J Geriatr Psychiatry.

[12] Tombaugh TN (2004). Trail Making Test A and B: normative data stratified by age and education. Arch Clin Neuropsychol.

[13] Wechsler Abbreviated Scale of Intelligence manual. Norwegian version. Bromma, Sweden: Pearson Assessment; 1999.

[14] Barca ML, Engedal K, Selbæk G (2010). A reliability and validity study of the Cornell scale among elderly inpatients, using various clinical criteria. Dement Geriatr Cogn Disord.

[15] Jorm AF (2004). The Informant Questionnaire on Cognitive Decline in the Elderly (IQCODE): a review. Int Psychogeriatr.

[16] Inouye SK, van Dyck CH, Alessi CA, Balkin S, Siegal AP, Horwitz RI (1990). Clarifying confusion: the Confusion Assessment Method: a new method for detection of delirium. Ann Intern Med.

[17] Delis DC, Kramer JH, Kaplan E, Ober BA (2004). California Verbal Learning Test—Second Edition (CVLT-II).

[18] Brunoni AR, Nitsche MA, Bolognini N, Bikson M, Wagner T, Merabet L (2012). Clinical research with transcranial direct current stimulation (tDCS): challenges and future directions. Brain Stimul.

[19] Freitas C, Mondragón-Llorca H, Pascual-Leone A (2011). Noninvasive brain stimulation in Alzheimer’s disease: systematic review and perspectives for the future. Exp Gerontol.

[20] Nardone R, Bergmann J, Christova M, Caleri F, Tezzon F, Ladurner G (2012). Effect of transcranial brain stimulation for the treatment of Alzheimer disease: a review. Int J Alzheimers Dis.

[21] Koch G, Di Lorenzo F, Bonnì S, Ponzo V, Caltagirone C, Martorana A (2012). Impaired LTP- but not LTD-like cortical plasticity in Alzheimer’s disease patients. J Alzheimers Dis.

[22] Tapia-Arancibia L, Aliaga E, Silhol M, Arancibia S (2008). New insights into brain BDNF function in normal aging and Alzheimer disease. Brain Res Rev.

[23] Elder GJ, Taylor JP (2014). Transcranial magnetic stimulation and transcranial direct current stimulation: treatments for cognitive and neuropsychiatric symptoms in the neurodegenerative dementias?. Alzheimers Res Ther.

[24] Kekic M, Boysen E, Campbell IC, Schmidt U (2016). A systematic review of the clinical efficacy of transcranial direct current stimulation (tDCS) in psychiatric disorders. J Psych Res.

[25] Carlesimo GA, Perri R, Caltagirone C (2011). Category cued recall following controlled encoding as a neuropsychological tool in the diagnosis of Alzheimer’s disease: a review of the evidence. Neuropsychol Rev.

[26] Cummings JL, Mega M, Gray K, Rosenberg-Thompson S, Carusi DA, Gornbein J (1994). The Neuropsychiatric Inventory: comprehensive assessment of psychopathology in dementia. Neurology.

[27] Sikkes SA, Knol DL, Pijnenburg YA, de Lange-de Klerk ES, Uitdehaag BM, Scheltens P (2013). Validation of the Amsterdam IADL Questionnaire©, a new tool to measure instrumental activities of daily living in dementia. Neuroepidemiology.

[28] Grill JD, Karlawish J (2010). Addressing the challenges to successful recruitment and retention in Alzheimer’s disease clinical trials. Alzheimers Res Ther.

[29] Bastani A, Jaberzadeh S (2014). Within-session repeated a-tDCS: the effects of repetition rate and inter-stimulus interval on corticospinal excitability and motor performance. Clin Neurophysiol.

[30] Nitsche MA, Kuo MF, Paulus W, Antal A, Knotkova H, Rasche D (2015). Transcranial direct current stimulation: protocols and physiological mechanisms of action. Textbook of neuromodulation: principles, methods and clinical applications.

